# Relationship between Heart Disease and Liver Disease: A Two-Way Street

**DOI:** 10.3390/cells9030567

**Published:** 2020-02-28

**Authors:** Hamza El Hadi, Angelo Di Vincenzo, Roberto Vettor, Marco Rossato

**Affiliations:** 1 Internal Medicine 3, Department of Medicine—DIMED, University of Padova, Via Giustiniani 2, 35100 Padova, Italy; dr.hamza.elhadi@gmail.com (H.E.H.); divincenzoang@gmail.com (A.D.V.); roberto.vettor@unipd.it (R.V.); 2 Department of Medicine, Klinikum Rheine, 48431 Rheine, Germany

**Keywords:** heart failure, arrhythmia, congestive hepatopathy, cardiogenic ischemic hepatitis, cirrhotic cardiomyopathy, liver transplantation, nonalcoholic fatty liver disease

## Abstract

In clinical practice, combined heart and liver dysfunctions coexist in the setting of the main heart and liver diseases because of complex cardiohepatic interactions. It is becoming increasingly crucial to identify these interactions between heart and liver in order to ensure an effective management of patients with heart or liver disease to provide an improvement in overall prognosis and therapy. In this review, we aim to summarize the cross-talk between heart and liver in the setting of the main pathologic conditions affecting these organs. Accordingly, we present the clinical manifestation, biochemical profiles, and histological findings of cardiogenic ischemic hepatitis and congestive hepatopathy due to acute and chronic heart failure, respectively. In addition, we discuss the main features of cardiac dysfunction in the setting of liver cirrhosis, nonalcoholic fatty liver disease, and those following liver transplantation.

## 1. Introduction

For many years, a mutual interaction between the heart and the liver has been established. Avicenna first pointed out the interactive effects between these two organs in his famous book titled as “Canon” (The Law). This interaction was described as dominance of the “heart warmth” over “liver coldness and wetness” and the dominance of “liver dryness” over “heart wetness”. According to traditional medicine, each organ is composed of four temperaments where the “wetness” and “dryness” are considered as a spectrum of “tissue moistures”,while “warmth” and “coldness” may be regarded as the basic metabolism of the organ. In normal condition, the heart’s temperament is “warm” and “dry” and the liver temperament is “warm” and “wet”. According to Avicenna, the presence of imbalancebetween the temperaments of human body describes the state of illness or organ disorder [1].

Both heart and liver diseases are regarded as a seriousburden on health system and a leading cause of deterioration of quality of life and shortened life expectancy. In this review, we discuss the complex cardiohepatic interactions in the setting of the main heart and liver diseases.

This review seeks to highlight how acute and chronic heart failure may lead to cardiogenic ischemic hepatitis and chronic congestive hepatopathy, respectively. In addition, this paper provides an overview on how chronic liver diseases including hepatic cirrhosis, nonalcoholic fatty liver disease (NAFLD), and conditions following liver transplantation (LT) may impair the cardiac performance and induce electrophysiological abnormalities in the absence of other cardiac disease. In each section, we discuss briefly the likely mechanisms underlying this association, clinical presentations, and diagnostic approaches.

## 2. The heart as a Cause of Liver Disease

### 2.1. Congestive Hepatopathy

**Figure 1:**
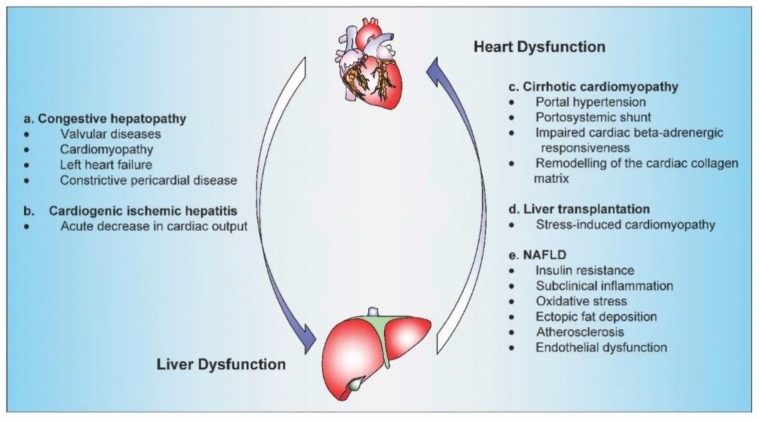
The suggested mechanisms underlying the cardiohepatic interactions in the setting of main heart and liver dysfunctions.(**a**) Congestive hepatopathy is most commonly observed in valvular heart diseases, cardiomyopathy, left heart failure, and constrictive pericardial disease. (**b**) An acute decrease in cardiac output may result in cardiogenic ischemic hepatitis. (**c**) In liver cirrhosis, the combination of portal hypertension, impaired cardiac beta-adrenergic responsiveness, and cardiac extracellular matrix remodeling isinvolved in the development of cirrhotic cardiomyopathy. (**d**) Stress cardiomyopathy is an acute heart failure syndrome that may appear in the perioperative period after liver transplantation. (**e**) Insulin resistance, subclinical inflammation, oxidative stress, ectopic fat deposition, atherosclerosis, and endothelial dysfunction are considered the main mechanisms linking NAFLD with cardiac complications.

Congestive hepatopathy or chronic passive hepatic congestion refers to the congestion of liver parenchyma induced by impaired hepatic venous outflow secondary to a right-sided cardiac failure (Figure 1a).

#### 2.1.1. Pathophysiology and Presentation

The underlying pathophysiological mechanisms include increased hepatic vein pressures, decreased hepatic blood flow, and decreased arterial oxygen saturation [2,3]. Main cardiac conditions associated with congestive hepatopathy include valvular diseases (tricuspid regurgitation and mitral stenosis), cardiomyopathy, left heart failure, and constrictive pericardial disease [4,5]. A growing population of patients at high risk for the development of chronic passive hepatic congestion is represented by adults with single ventricle congenital heart disease who have undergone surgical palliation with the Fontan’s procedure. This surgical procedure consists of connecting a single working heart ventricle to the systemic circulation while allowing passive venous return to the pulmonary arteries. Over time, central venous pressure increases and cardiac output decreases resulting in severe hepatic congestion [6].

Congestive hepatopathy is usually subclinical. When symptomatic, patients may present with early satiety, malaise, mild jaundice, or intermittent right upper quadrant pain secondary to dilatation of the liver capsule. Physical examination is typically dominated by signs of cardiac failure including jugular vein distension, hepatojugular reflux, and peripheral edema. Spider angiomata, splenomegaly, and varices are rarely present [5,7]. The presence of esophageal varices indicates an elevated transhepatic pressure gradient due to progression toward liver fibrosis [8]. In addition, presence of pulsatile liver can be noticed in the setting of tricuspid valve regurgitation. The loss of this pulsatility over time suggests the progression from congestive hepatopathy to secondary liver cirrhosis known as cardiac cirrhosis, a term grouping all the hepatic disorders that occur secondary to hepatic congestion due to cardiac dysfunction, mainly the right heart [9].

#### 2.1.2. Biochemical Pattern

Primary laboratory finding in congestive hepatopathy is represented by mild hyperbilirubinemia, with a predominantly unconjugated fraction [3]. Elevations in markers of cholestasis (alkaline phosphatase (ALP) and gamma-glutamyltransferase (GGT) are also characteristic of congestive hepatopathy and have been shown to correlate with right atrial and central venous pressures [10,11].

Mild hypoalbuminemia and ascites may be present in patients with congestive hepatopathy. In these patients, the state of inadequate nutrition and hemodilution due to heart failure contributes substantially to the aggravation of hypoalbuminemia [12].

#### 2.1.3. Histopathology

Morphologically, congestive hepatopathy is associated with characteristic changes within the liver parenchyma [3,13,14]. Elevated hepatic venous pressure is transmitted to lobular hepatic vessels inducing an enlargement of sinusoidal fenestrae [5]. The dilatation of hepatic sinusoids is correlated with degree of venous pressure and results in the exudation of red blood cells and protein rich-fluid into the perisinusoidal space of Disse [5]. Prolonged perivenular congestion induces the so-called cardiac liver cirrhosis characterized by local steatotic changes, atrophy, and/or necrosis within the centrilobular hepatic parenchyma (zone 3). Over time, this may progress in deposition of sinusoidal collage to form broad fibrous septa that extend between adjacent central hepatic veins [15]. This pattern of bridging fibrosis produces a typical histologic aspect referred as “reverse lobulation” [16]. The degree of fibrosis varies within the spatial regions of the livers. This heterogeneity is likely related to the fibrogenic effects of focal thrombotic events within the hepatic venules and sinusoids due to the chronic vascular congestion. The gross pathological appearance of a liver affected by chronic venous congestion has been described as “nutmeg liver”. This speckled appearance is due to the extravasation of red blood cells from the centrilobular sinusoids, which leads to reddened areas within the hepatic parenchyma [17].

#### 2.1.4. Management

Treatment of congestive hepatopathy consists mainly of treating the underlying cardiac disease and improvement of cardiac output. Diuretics represent the main stay of pharmacological therapy. Unless contraindicated, angiotensin-converting enzyme (ACE) inhibitors and beta blockers are indicated in symptomatic congestive heart failure. A surgical approach may be indicated in the case of constrictive pericarditis, severe valvular heart disease, and ischemic cardiomyopathy [9,18]. For patients with end-stage heart failure, temporary left ventricular assistive device support (LVADs) or cardiac transplantation may help to improve congestive hepatopathy [19,20].

### 2.2. Cardiogenic Ischemic Hepatitis

Cardiogenic ischemic hepatitis, known also as acute cardiogenic liver injury, is a clinical and histological syndrome characterized by a rapid and transient rise in serum transaminase levels due to acute fall in cardiac output leading to the reduction of hepatic blood flow [21] (Figure 1b).

#### 2.2.1. Pathophysiology and Presentation

Cardiogenic ischemic hepatitis has been estimated to emerge in 20–30% of patients with acute heart failure [22]. The pathophysiological mechanisms involved in this acute liver damage are mainly related to changes of hepatic blood flow. In physiological conditions, hepatic blood flow accounts for about 20% of the cardiac output, delivered to the liver via the arterial hepatic system and the portal vein. Reduced portal venous flow, due to a decrease in cardiac blood flow, leads to a rise in adenosine levels which is synthesized by liver cells and Kuppfer cells. The increased adenosine levels induce an apparently protective hepatic artery dilatation via an autoregulatory mechanism known as “hepatic arterial buffer response” [23]. Nevertheless, when hypotension is persistent, visceral flow is critically reduced and severe hypoxemia develops, leading to hepatocellular hypoxia and necrosis after overwhelming the compensatory mechanisms [24].

An acute cardiogenic liver injury is usually related to an acute coronary event, cardiac arrhythmia, or transient severe hypotension [25]. Findings from a retrospective study demonstrated that acute decline in hepatic perfusion during cardiogenic shock is not the sole incident underlying the development of acute liver injury. The analysis proposed that hepatic venous congestion due to right-sided heart failure predisposes to acute hepatic injury induced by hypotensive event [21].

In another report of patients with clinical and biochemical evidence of acute liver injury admitted to the coronary intensive care unit, the presence of acute liver injury has been shown to be associated with a decrease in hepatic blood flow and passive hepatic venous congestion [24]. Additionally, Birrer et al. showed that systemic vascular resistance and mean pulmonary capillary wedge pressure were elevated in patients with acute hypoxic liver injury [26]. Taken together, these findings suggest that the combination of hepatic venous congestion coupled with acute cardiac event is required for the development of acute cardiogenic liver injury.

Acute cardiogenic hepatitis is usually asymptomatic. However, clinical signs may appear after a latency period of 2–24 h. Most commonly, patients exhibit symptoms resembling acute viral hepatitis such as weakness, apathy, and, in a minority of cases, mental confusion, flapping tremor, oliguria, jaundice, and hepatic coma [25,27].

#### 2.2.2. Biochemical Pattern

The diagnostic approach of cardiogenic ischemic hepatitis is usually based on clinical presentation and laboratory finding. Non-invasive imaging as abdominal ultrasound can be supportive by showing signs liver congestion as dilation of the suprahepatic veins and inferior vena cava [28]. Pulmonary hypertension and impaired systolic left ventricular function are frequently observed by echocardiography. Other imaging techniques such as magnetic resonance or computed tomography or imaging, may be implied to exclude other causes of liver injury [28].

The typical pattern in laboratory tests consists of rapid, transient rises in serum aminotransferase (aspartate [AST] and alanine aminotransferase [ALT]) and lactate dehydrogenase levels (LDH) with peak levels reaching between 10 and 20 times the upper limit of normal [3,29].

The ratio of serum ALT to lactate LDH less than 1.5 early in the course of liver injury is characteristic of cardiogenic injury [30]. After correction of the underlying process, these liver enzymes drop gradually and reach normal values within 7–10 days [3]. Other non-specific laboratory abnormalities include an elevation in total serum bilirubin levels. In addition, International Normalized Ratio (INR) can be prolonged due to deficiency of liver coagulation factors [31].

#### 2.2.3. Histopathology

The main histologic feature of acute cardiogenic ischemic hepatitis is characterized by hepatic necrosis around the central hepatic veins (zone 3), a condition known as centrilobular necrosis. This zone has the poorest oxygen delivery, and will be most affected during a low hepatic blood flow [3]. In cases of prolonged circulatory collapse, necrotic changes may extend to midzonal periportal segments (zone 2) [3,32].

#### 2.2.4. Management

The management of cardiogenic ischemic hepatitis focuses on identifying and removing the precipitating cause. This may include medications with hypotensive or negative inotropic effects and other medications that likely may induce impairment of renal function.

When needed, administration of oxygen and positive inotropic agents may help to restore an adequate organ perfusion [33].

## 3. The Liver as a Cause of Heart Disease

### 3.1. Cirrhotic Cardiomyopathy

Cirrhotic cardiomyopathy (CCM) was first described in the late 1960s, although for many years, it was mistakenly presumed to result from a latent alcoholic cardiomyopathy [34]. CCM represents a clinical entity in patients with cirrhosis characterized by a combination of impaired myocardial contractile responsiveness to stress, diastolic dysfunction along with electrophysiological disturbances, in the absence of other known cardiac disease [35,36]. Although the diagnosis of CCM is usually missed or delayed, it was estimated that this condition can be present in up to 50% of patients with cirrhosis (Figure 1c) [37].

#### 3.1.1. Pathophysiology and Presentation

##### Hyperdynamic Circulation

In hepatic cirrhosis, portal hypertension results in splanchnic arterial vasodilation due to the release of potential liver-derived vasodilators factors such as carbon monoxide, nitric oxide, and prostacycline [38,39]. This leads to reduced systemic vascular resistance, low arterial blood pressure, increased splanchnic blood redistribution, and central hypovolemia [40,41]. The state of central hypovolemia is also enhanced through the portosystemic shunting and bacterial translocation. Taken together, these processes induce an activation of sympathetic nervous system with consequent hyperdynamic circulation due to an increase of heart rate and cardiac output [42].

##### Systolic Dysfunction

Patients with liver cirrhosis present an impaired ventricular ejection of fraction under physical stress compared with non-cirrhotic subjects. This is mainly related to inadequate heart rate response and decreased myocardial contractility under exercise [43,44].

Alterations in β-adrenergic receptor function at the membrane of cardiac muscle cells have beenshown to be involved in the myocardial hyporesponsiveness of cirrhotic patients. It is well accepted that the long-term exposure of cardiomyocytes to high levels of noradrenalin usually present in cirrhosis due to enhanced sympathetic tone results in internalization, sequestration, and down regulation of β-adrenergic receptors on plasma membrane [45,46].

Experimental findings from animal models suggested that nitric oxide (NO) could play a negative inotropic effect in heart of cirrhotic rats [47]. The activation of NO-pathway in heart tissue of cirrhotic rats has been shown to be at least in part related to the stimulation of inducible NO synthetase (iNOs)through the elevated levels of proinflammatory cytokines such as interleukin 1 beta (IL-1β) andtumor necrosis factor alpha(TNF-α) [48]. In this regard, the endocannabinoid system has been shown to contribute to the decreased contractility of cardiomyocyte in experimental liver cirrhosis [49]. The increased endotoxemia which may occur in liver cirrhosis has been shown to enhance the production of macrophage-, lymphocyte- and platelet-derived endocannabinoids [50].

In this context, arachidonoylethanolamide (AEA) or anandamide and 2-arachidonoylglycerol (2-AG) are the most widely studied endocannabinoids, exerting their effects via G protein-coupled cannabinoid receptors (CBRs). The activation of cannabinoid receptor type 1 (CB-1) has been shown to decrease the contractile response to β-adrenergic agonists. This cardiac adrenergic responsiveness was restored by blockage of CB-1 receptor [51]. In addition, elevated cardiac levels of anandamide have been shown to mediate at least in part the inhibitory effects of TNF-α on cardiac contractility [52].

##### Diastolic Dysfunction

Abnormalities of diastolic dysfunction are considered an early manifestation of CCM, and frequently precede the systolic dysfunction. Left ventricular diastolic dysfunction is characterized by a decrease in the rate and degree of left ventricular relaxation, together with presence elevated end-diastolic filling pressures [53]. Unlike contractile systolic failure, which usually appears under stress conditions, echocardiographic findings of diastolic dysfunction can also manifest at rest [54]. However, its underlying mechanisms remain to be fully elucidated. In a rat model of liver cirrhosis, alterations in titin protein modulation have been shown to be an important determinant of diastolic stiffness in CCM. Titin is a giant multi-functional sarcomeric filament that provides passive stiffness to cardiomyocytes and thus responsible for the elasticity of the relaxed striated muscle [55]. Moreover, it was hypothesized that abnormalities in the cardiac β-adrenergic signaling pathway induce a decrease in protein kinase A (PKA) levels that may ultimately impair phosphorylation of titin and subsequently increase passive tension of left ventricle. Additionally, a reduced level of PKA induces a decrease in PKA-dependent cardiac troponin I (cTnI) phosphorylation, which leads to decreased dissociation of calcium from cardiac troponin C (cTnC) and thus may alter rate of cardiac muscle relaxation [56].

Liver cirrhosis was associated with changes in expression of collagen isoforms in ventricular heart tissue has been shown to be involved to correlate with diastolic dysfunction in cirrhosis. Ventricular cardiac tissue derived from cirrhotic rats showed an increased collagen type 1 content compared with sham animals, which subsequently contributes significantly to diastolic dysfunction by increasing the passive cardiac stiffness [55].

##### Electrophysiological Modifications

A prolonged QT interval represents a common electrocardiographic finding in patients with cirrhosis and served as predictor of the increased risk of ventricular arrhythmia and/or sudden death [57]. QT prolongation has been attributed to the downregulation of β-adrenergic receptors after chronic exposure to noradrenalin [58]. In addition, elevated bile salts as well as uric acid plasma level have been shown to predispose to prolonged QT intervals in cirrhotic patients [59].

Chronotropic incompetence is another consistent finding in CCM, and refers to the inability of the heart to increase adequately its rate to match the metabolic demands. Cardiovascular autonomic dysfunction has been suggested to play a role in enhancing chronotropic incompetence in patients with CCM [60].

#### 3.1.2. Diagnostic Approach

Considering the subclinical picture of CCM in the majority of patients, the diagnostic approach involves a combination of laboratory, clinical, electrocardiographic, and imaging evaluations. Electrocardiographic assessments may be helpful to identify cirrhotic patients with prolongations of QT interval and arrhythmias. Echocardiographic evaluation of the left ventricular systolic function usually shows a reduction of ventricular ejection of fraction at rest or under stress in response to catecholamine stimulation in the case of subtle systolic dysfunction. Diastolic impairment in CCM manifests mainly by reduced early (E) ventricular filling capacity and increased atrial (A) filling (E/A<1.0), in addition to prolongation of deceleration time (>200 ms) and isovolumetric relaxation time (ITVR > 80ms) [61].

Biomarkers for cardiac dysfunction (cTnI, B-type natriuretic peptide [BNP], and [pro-BNP]) might be elevated in early stages of CCM and usually reflect the severity of liver disease [62].

#### 3.1.3. Management

CCM complicates several therapeutic approaches used in cirrhosis and heart failure. Although beta blockers are contraindicated in decompensated cirrhosis with ascites, these drugs have been shown to normalize the prolonged QT interval and might reduce the hyperdynamic load [63]. ACE inhibitors and angiotensin receptor blockers (ARBs) are contraindicated in presence of ascites because of the risk of hypotension and hepatic-renal syndrome [63]. LT may be an effective treatment forend-stage liver disease associated with CCM since it has been shown to reverse cardiac dysfunctionand electrophysiological abnormalities [63]. However, many cardiac complications have been reported in patients with CCM undergoing LT [64].

### 3.2. Liver Transplantation

Cardiovascular events and mainly heart failure represent a primary source of morbidity and mortality following LT. Heart failure may have an early-onset (within 30 days after transplantation) or in other cases may occur late (>30 days). In the postoperative period, heart failure may reach an overall incidence of 3.3% and may be associated with a mortality risk of 45% [65] (Figure 1d).

#### Stress-Induced Cardiomyopathy

##### Clinical Presentation

The cardiovascular stress due to surgery is considered a main contributor of early heart failure in liver transplanted patients. Takotsubo cardiomyopathy (TTC) or stress-induced cardiomyopathy represents a main cause of acute heart failure in the absence of coronary artery disease [66]. TTC’s clinical pattern is distinctive since it is characterized by a transient hypo- or dyskinesia of the apical, lateral, and posterior wall of left ventricle and a preservation of the basal cardiac segments [67,68]. Even though the clinical presentation is often dramatic and accompanied by cardiogenic shock and lethal arrhythmias, the recovery is usually rapid and complete [69,70]. Even though TTC occurs in the absence of angiographic evidence of coronary artery disease, it is often difficult to differentiate this entity from an ischemic event related to coronary thrombosis.

##### Pathophysiology

Several pathophysiological mechanisms have been suggested to be involved in TTC. The exposure of myocardium to large concentrations of circulating catecholamines during LT makes the heart susceptible to adverse events including multivessel epicardial vasospasm [71]. In addition, the altered adrenergic and Gs protein signaling pathways that are commonly found in heart tissue of cirrhotic patients have been suggested to contribute in TTC physiology [72].

Intraoperative triggers for TTC during liver transplantation surgery include cardiovascular stress, such uncontrolled bleeding, massive transfusions, and administration of high rates of vasoactive agents [73].

##### Predictors and Diagnostic Approach

Stress-induced cardiomyopathy is still an underestimated cause of acute heart failure in liver transplanted patients. Beside the exclusion of coronary disease, the diagnostic approach should involve the combination of clinical aspects to electrocardiographic and echocardiographic findings. Multiple studies have evaluated the risk factors for late cardiac events following LT. The severity of liver disease as quantified by Model of End Stage Liver Disease (MELD) score has been shown to be associated with adverse cardiac events following LT [74]. This was attributed to presence CCM and its components including diastolic dysfunction and prolonged QT interval [75,76].

In addition to older age and male sex, several components of the metabolic syndrome factors including hypertension, hyperglycemia, dyslipidemia, and visceral obesity have been shown to be predictive for heart failure following LT [77,78].In this context, elevated pre-transplantation blood urea nitrogen plasma levels and hemodialysis increase the risk of postoperative heart failure [79] Similarly, pulmonary hypertension and presence of history for cardiac disease including arrhythmia and coronary artery disease have been shown to predict post-transplantation cardiac complications [74,77]. Currently, LT candidates undergo a preoperative systemic assessment to improve the post-transplantation outcome. This includes a full history and examination, 12-lead ECG, and two-dimensional echocardiography to detect structural and functional heart abnormalities and estimate pulmonary artery pressure. Basedon the findings of the initial screening test, liver transplant recipients who are at high-risk for coronary artery disease undergo further invasive investigations such as dobutamine stress echocardiography, coronary angiography, and coronary computed tomography angiography (CCTA) [80].

##### Management

Hemodynamically stable patients are often treated with diuretics, ACE inhibitors, and beta blockers. Treatment with calcium channel blockers and fluid resuscitation is usually indicated for patients with severe left ventricular outflow tract obstruction and hemodynamic compromise. Inotropic agents can be administered only in absence of moderate or severe left ventricular outflow tract obstruction. In presence of TTC with thromboembolism, anticoagulation should be continued for at least three months [81].

### 3.3. Nonalcoholic Fatty Liver Disease

#### 3.3.1. Definition and Prevalence

NAFLD encompasses a pathological condition characterized by ectopic deposition of adipose tissue in the liver, in the absence of secondary causes for hepatic fat accumulation, such as excessive alcohol intake, steatogenic drugs, and hereditary disorders [82]. This terminology covers a wide spectrum that ranges from simple steatosis or nonalcoholic fatty liver (NAFL) to nonalcoholic steatohepatitis (NASH) and cirrhosis. Hepatic steatosis is considered a benign and reversible stage characterized by excessive fat infiltration involving more than 5% of the liver parenchyma, with no evidence of hepatocyte injury. NASH is characterized by steatosis with hepatocellular inflammation that may further progress in liver fibrosis, cirrhosis, and hepatocellular carcinoma [83].

NAFLD is the most common chronic liver disease worldwide with a prevalence of about 20–30% in the general population and it is estimated to be 50–90% in people with obesity and type 2 diabetes mellitus. Today, the global prevalence of NAFLD mirrors the steady increase in obesity rate and correlates with its degree. Hepatic steatosis is found in 65% of subjects with grade I–II obesity (BodyMass Index [BMI] = 30–39.9 kg/m^2^) and in 85% of patients with morbid obesity (BMI = 40–59 kg/m^2^) [84]. There is large growing evidence suggesting that NAFLD contributes potentially to the increased risk for cardiovascular-related morbidity and mortality [85,86]. According to the Framingham scoring approach, the mean of respective cardiovascular risks in subjects with and without NAFLD was estimated as 16.0% and 12.7% in men and 6.7% and 4.6% in women, respectively [87].

#### 3.3.2. Cardiac Disorders in NAFLD

The most common cardiac complications associated with NAFLD include coronary artery disease, and structural myocardial alterations in addition to cardiac arrhythmias. An increasing number of studies have addressed a link between coronary heart disease and NAFLD independently of traditional cardiovascular risk factors [88]. Patients with NAFLD showed a higher prevalence of calcified and non-calcified coronary plaques compared to healthy individuals independently of the presence of the metabolic syndrome components [89].

Moreover, the coronary microvascular and endothelial function assessed by the measurement of coronary flow reserve (CFR) has been shown to be impaired in patients with NAFLD compared with healthy subjects [90].

Moreover, subjects with NAFLD have been shown to have common features of left ventricular diastolic dysfunction, which represents a key contributor to the development of heart failure [91]. Similarly, a higher prevalence of left ventricular mass index compared to controls has been observed [92]. Furthermore, NAFLD has been associated with morphological and valvular heart abnormalities. Several studies showed that hepatic steatosis is associated with aortic-valve sclerosis and mitral annular calcification, both known to enhance the risk of cardiac arrhythmias and heart failure [93,94].

Cardiac rhythm disturbances are also linked to NAFLD, probably due to an induction of cardiac autonomic dysfunction. Several arrhythmias are found in association with NAFLD such as atrial fibrillation, QT prolongation, and ventricular arrhythmias, which can predispose these patients to sudden cardiac death [95].

#### 3.3.3. Mechanisms Linking Cardiac Disorders to NAFLD

The precise mechanisms linking NAFLD with cardiac complications are poorly understood. However, several pathophysiological mechanisms have been postulated to be involved such as insulin resistance, visceral adiposity, subclinical inflammation, dyslipidemia, and oxidative stress (Figure 1e) [96].

NAFLD patients tend to present whole-body as well as hepatic insulin resistance, inducing disturbances in the transport of free fatty acids, thus leading to visceral and ectopic fat accumulation. Due to the increased lipogenesis, the lipid profile in these subjects consists in increased levels of triglyceride, low-density lipoprotein cholesterol (LDL-C), and very low-density lipoprotein cholesterol (VLDL-C), which are consequently linked to increased cardiovascular risks [97,98].

NAFLD is considered a chronic subclinical inflammatory condition through secreting proinflammatory cytokines and chemokines by both hepatocytes and by non-parenchymal cells such as stellate cells and Kupffer cells [99]. Several inflammatory markers as well as serum levels of IL-6, IL-1, TNF-α, and C-reactive protein (CRP) have been demonstrated to be related to cardiovascular events in NAFLD [100].

In addition to the role of abdominal obesity, which usually occurs in NAFLD, ectopic fat accumulation in other districts (i.e. perivascular, pericardial, and epicardial) leads to adipocytes dysfunction with consequent imbalance of pro- and anti-inflammatory cytokinesoriginating from adipose tissue termed adipokines [101,102]. In this context, an increase in proinflammatory cytokines such as leptin and a decrease of cardioprotective one such as adiponectin have been reported in NAFLD [102]. This induces a persistent deterioration of the inflammatory and insulin resistant states that consequently lead to the worsening of cardiometabolic outcomes [101].

Oxidative stress appears to play a key role in promoting the progression from simple steatosis to NASH and increasing the cardiometabolic risk in NAFLD patients [103]. It arises as a consequence of an imbalance between excessive generation of reactive oxygen species (resulting from accelerated FFA influx and de novo lipid synthesis) and antioxidant defenses leading to a disruption of redox signaling and molecular damage [103]. Oxidative stress enhances the atherosclerotic process by inducing an endothelial dysfunction and subsequently predisposes NAFLD subject to cardiovascular events [104]. In addition, it leads to impairment in mitochondrial function, which is associated with both insulin resistance and atherosclerosis [105].

A high production of prothrombotic factors, mainly factors VII, IX, XI and XII, has been shown to be linked to NAFLD [106] and therefore can contribute to an increase in cardiovascular risk and atherosclerosis in these patients [107]. In the same context, increased evidence supports the positive relationship between circulating levels of plasminogen inhibitor activator 1 (PAI-1) and hepatic fat ontent and NAFLD severity [108]. An increased release of the PAI is known to be a good predictor of ischemic heart disease [109].

#### 3.3.4. Predictors of Cardiac Events in NAFLD

In recent years, novel hepatic tissue-specific molecules known as hepatokines have been described to determine cardiovascular effects. Fibroblast growth factor 21 (FGF-21) is a peptide predominantly produced and secreted by the liver and correlates with hepatic fat content and the severity of NAFLD [110]. Elevated FGF-21 serum levels have been shown to be associated with atherosclerosis [111], and coronary heart disease [112]. NAFLD has been related with higher serum levels of another hepatokine known as Fetuin-A (FetA) [113]. FetA plasma levels have been shown to be positively related with negative cardiovascular outcomes as well as endothelial dysfunction and myocardial infarction [114].

Given the rapidly growing global burden of NAFLD and limitations of performing liver biopsy due to interobserver variability and procedure-related morbidity and mortality, recent recommendations suggested several noninvasive markers of fibrosis and steatosis [115]. The NAFLD Fibrosis Score (NFS) is a simple, serum-based index, originally developed for the diagnosis of advanced hepatic fibrosis in patients with NAFLD representing an independent predictor for overall cardiovascular events and mortality, including cardiac death [116].

#### 3.3.5. Management

The main clinical recommendations for the management of NAFLD include lifestyle modifications through a combination of physical activity, smoking cessation, appropriate dietary changes, and gradual weight loss. This approach has demonstrated to be an effective tool to improve the cardiovascular risk profile in NAFLD patients.

Moreover, current guidelines recommend careful clinical screening and treatment for associated cardiometabolic risk factors including obesity, diabetes mellitus, dyslipidemia, and hypertension in all individuals with NAFLD. Bariatric surgery represents a recommended choice for morbidly obese individualsif lifestyle modifications and pharmacological approach have not yielded long-term success. It provides cardiovascular benefits in terms of reducing overall mortality by improving hepatic inflammation, metabolic syndrome components, and cardiac morphology [96,117].

## 4. The Heart–Liver Axis

Increasing evidence suggests that the heart synthesizes and secretes proteins referred to as cardiokines, which are involved in the inter-cellular and inter-organ communication [118]. More than 16 cardiokines have been identified, including the atrial natriuretic factor (ANF), BNP, transforming growth factor-β1 (TGF-β1), angiotensin II, and proinflammatory cytokines which are known to play physiological and pathological role in cardiac fibrosis, apoptosis, and metabolism [119].

It has been reported that cardiokinesnot only have local involvement in the stress response and damage repair, but also mediate several regulatory effects on extra-cardiac tissues [120]. In the same context, ANP, a well know cardiokinesecreted from the cardiac tissue and known to mediate fluid homeostasis in heart failure, has been shown to attenuate glycolysis and increased gluconeogenesis in rat liver [121].

NOD-like receptor protein 3 (NLRP3) inflammasomeis the most widely studied inflammasome in myocardial tissue. It consists of a multi-protein signaling platform that acts as an intracellular innate immune sensor and its activation induces the transcription, maturation, and release of proinflammatory cytokines, in particular IL-1β and IL-18. In heart failure, three main triggers contribute to the activation of NLRP3 inflammasome components: adenosine triphosphate (ATP), mitochondrial DNA (mtDNA), and reactive oxygen species (ROS) [122]. Despite the clear evidence that cardiokinesare involved in the crosstalk between myocardial inflammation in heart failure and peripheral tissue damage in some organs (adipose, tissue, skeletal muscle, spleen, and kidney), direct mechanisms linking heart and liver disease remain not fully characterized. In animal model, congestive heart failure has been shown to be associated with altered metabolism and proinflammatory response in liver [123]. In this study, the serum concentration and hepatic gene expression of proinflammatory cytokines TNF-α andIL1-βsignificantly increased in the case of congestive heart failure. In addition, blood levels of hepatic proteins, such as albumin, transthyretin, transferrin, and retinol-binding protein, were decreased. In congestive heart failure rats, the gene expression of enzymes related to lipogenesis was increased, while that related to gluconeogenesis was decreased. In addition, during fasting, the liver of heart failure rats showed a maladaptive response by incorporating more glucose instead of releasing energy substrates [123], probably because of state of liver inflammation. 

Olson’s group reported that the heart controls systemic energy metabolism, fat mass, and body weight via the heart specific microRNA-208a (miR-208a) and Mediator complex subunit 13 (MED13) signaling in rodent cardiomyocytes [124]. MiR-208a is encoded by an intron of the α-cardiac muscle myosin heavy chain gene (Myh6) and is expressed specifically in the heart. MiR-208a is mainly involved in cardiac remodeling and regulation of cardiac hypertrophy pathway. MED13 is a mediatorof transcription machineryand contributes to cardiac and systemic metabolism by controlling gene expression and mitochondrial numbers through thyroid hormone receptors and other nuclear hormones [125]. Overexpression of MED13 or inhibition ofmiR-208a in cardiac tissue of transgenic mice has been shown to enhance lipid uptake, β-oxidation, mitochondrial content, and other genes involved in fatty acids utilization in adipose tissue and liver. These findings supportthe existence of a metabolic crosstalk between heart and liver [126].

The co-existence of bidirectional communication was also confirmed in patients with heart failure [127]. Impaired left ventricular function was associated with a decrease of endogenous hepatic unsaturated fatty acids circulatory levels. It was hypothesized that an increased left ventricular wall stress and pulmonary pressure can be transmitted to the liver veins and cause hepatic congestion and consequently may alter the fatty acid profile in the liver [127]. On the other hand, reduced levels of unsaturated fatty acids were correlated with adverse cardiac consequences, probably by interfering with proinflammatory processes such as nuclear factor κB (NF-κB) activation and TNF-α release [127,128].

## 5. Conclusions

In this review paper, we highlight how acute and chronic heart failure may lead to acute hepatic injury and chronic congestive hepatopathy, clinical conditions that are often accompanied by specific clinical and laboratory manifestations. On the other hand, subjects with liver diseases, mainly hepatic cirrhosis and NAFLD, or those undergoing LT can develop heart systolic and diastolic dysfunctions in addition to electrophysiological cardiac abnormalities. In this regard, a better knowledge of the cardiohepatic interactions constitutes a key issuefor the management of cardiac and hepatic dysfunctions.

Although still challenging, especially in multi-morbid patients, the early recognition of clinical signs and symptoms of simultaneous heart and liver injury has led to important benefits in terms of reduction of morbidity and mortality. This requires the involvement of a multidisciplinary approach to patients in order to manage the patients at early disease stages. Future research will unravel the molecular mechanisms implicated in the cardiohepatic interactions to further improve the diagnosis and therapy of these disturbances.

## Abbreviations

2-AG	anandamide and 2-arachidonoylglycerol
ACE	angiotensin-converting enzyme
AEA	Arachidonoylethanolamide
ALP	alkaline phosphatase
ALT	alanine aminotransferase
ANF	atrial natriuretic factor
ARBs	angiotensin receptor blockers
AST	aspartate aminotransferase
ATP	adenosine triphosphate
BMI	Body mass index
BNP	B-type natriuretic peptide
CB-1	cannabinoid receptor type 1
CBRs	cannabinoid receptors
CCM	cirrhotic cardiomyopathy
CCTA	coronary computed tomography angiography
CFR	coronary flow reserve
CRP	C-reactive protein
cTnC	troponin C
cTnI	troponin I
FetA	Fetuin-A
FGF21	fibroblast growth factor 21
GGT	gamma-glutamyltransferase
Il-1 β	interleukin 1 beta
iNOs	nitric oxide synthetase
INR	International Normalized Ratio
ITVR	isovolumetric relaxation time
LDH	lactate dehydrogenase
LDL-C	low-density lipoprotein cholesterol
LT	liver transplantation
LVAD	left ventricular assistive device support
MED13	mediator complex subunit 13
miR-208a	microRNA-208a
mtDNA	mitochondrial DNA
Myh6	myosin Heavy Chain 6
NAFL	nonalcoholic fatty liver
NAFLD	nonalcoholic fatty liver disease
NASH	nonalcoholic steatohepatitis
NFS	NAFLD Fibrosis Score
NF-κB	nuclear factor κB
NLRP3	NOD-like receptor protein 3
NO	nitric oxide
PAI-1	plasminogen inhibitor activator 1
PKA	protein kinase A
ROS	reactive oxygen species
TGF-β1	transforming growth factor-β1
TTC	Takotsubo cardiomyopathy
VLDL-C	low-density lipoprotein cholesterol
